# Relationship between RBC Mercury Levels and Serum n3 Polyunsaturated Fatty Acid Concentrations among Japanese Men and Women

**DOI:** 10.1155/2012/849305

**Published:** 2012-01-12

**Authors:** Mayumi Tsuji, Tetsuo Ando, Takao Kitano, Junji Wakamiya, Chihaya Koriyama, Suminori Akiba

**Affiliations:** ^1^Department of Environmental Health, University of Occupational and Environmental Health, 1-1 Iseigaoka, Yahatanishi-ku, Kitakyusyu 807-8555, Japan; ^2^Department of Epidemiology and Preventive Medicine, Kagoshima University Graduate School of Medical and Dental Sciences, 8-35-1 Sakuragaoka, Kagoshima 890-8544, Japan; ^3^Department of Public Health, Graduate School of Medical Sciences, Kumamoto University, 1-1-1 Honjo, Kumamoto City 860-8556, Japan

## Abstract

*Aims*. To evaluate potential health risk and benefits of fish consumption, the association of fish consumption with total mercury levels in red blood cells (RBCs) and serum eicosapentaenoic acid (EPA) concentrations was examined. *Subjects and Methods*. Study subjects were 269 Japanese (98 men and 171 women) living in a remote island of Kagoshima, and their blood was drawn in 1994. *Results*. Total mercury levels were related to weekly fish consumption among women (*P* = 0.035) but not among men (*P* = 0.643). However, serum EPA levels were not related to fish consumption in both women and men. In contrast, EPA levels in the high-density ipoprotein (HDL) fraction of the sera were significantly related to fish consumption (*P* values for men and women were 0.014 and 0.073, resp.). Interestingly, mercury levels were related to serum EPA levels and EPA in the HDL fraction of the sera (*P* = 0.001) among women (*P* = 0.005) but not among men. Sex differences in fish species consumed may be an explanation for the observed sex difference. *Conclusion*. Those findings suggest that the health benefit of fish consumption can be maximized by the careful selection of fish species consumed.

## 1. Introduction

Fish consumption increases the intake of n3 series polyunsaturated fatty acids (PUFAs), including eicosapentaenoic acid (EPA) and docosahexaenoic acid (DHA), which are reported to have health benefits. For example, it has been reported that n3-PUFA may decrease blood pressure [1] and ischemic heart disease risk [2, 3]. On the other hand, fish consumption is associated with methyl mercury intake [4–6], which may cause adverse health effects. However, the amount of methyl mercury intake through fish consumption is not necessarily proportional to n3-PUFA since methyl mercury concentration in fish meat is higher in fish species high on the marine food chain while n3-PUFA concentration in fish meat is not related to it [7, 8]. This study examined the association of fish consumption with blood mercury and serum EPA levels in order to examine potential health risk and benefits of fish consumption.

## 2. Materials and Methods

In July of 1994, the nutrition interview survey for over sixties was conducted, which was a part of medical checkup for elderly people in K town of a southern prefecture in Kyusyu, Japan. We collected the information on the frequencies of consumption of various food items, including raw fish, fish paste products, and dry fish. 

During the health checkup, blood samples were also obtained from the subjects and separated into the serum elements and the red blood cells (RBC) by centrifugation within a day. These blood specimens were stored at −20°C until use for the measurements of fatty acid compositions and the total mercury concentrations. 

Methyl-esterified fatty acids from either the total serum or the serum high-density lipoprotein (HDL) fractions separated from the total serum by the precipitated method of dextran sulphate Mg^++^ [9] were analyzed by gas liquid chromatography on 5% Shinchrome E71 [10]. EPA (C 20:5) in the total serum was measured. EPA was also measured in the serum HDL fraction to examine its long-term consumption. Serum EPA was 64.5 mg/L. EPA in HDL fraction was 26.2 mg/L. Therefore, 40.6% of EPA would be included in HDL fraction.

Total mercury concentration of RBC was measured by the cold vapor atomic absorption spectrometry after wet digestion in 2.5 mL sulfuric acid and 1 mL mixture of nitric and perchloric acids (5:1:1) at 250°C for 20 minutes [11]. Quality control of mercury analysis was conducted by comparing with the analysis of reference standard (e.g., IAEA085 and 086). Calibration of fatty acid measurements was conducted by comparison with the relative retention time of each fatty acid to septadecanoic acid (C 17:0, 100 mg/L), which was used as an internal standard on GC. Authentic fatty acid-methyl esters were obtained from fish oil on silica-gel thin-layer chromatograph impregnated 20% of silver nitrate by hexane-ethyl-ether (90:10) as a solvent system. Each authentic fatty acid was analyzed on GC with C 17:0 to obtain each relative retention time to C 17:0.

## 3. Statistical Analysis

Geometric means and corresponding 95% confidence intervals for the concentrations of total mercury in RBCs (RBC Hg) and EPA in the serum were calculated. Univariate and multivariate regression analyses were conducted by the intercooled Stata 8.1. Since the distributions of RBC Hg and EPA were skewed and had a long upper tail, log-transformed values were used in these analyses. Trend of association was assessed by regression models assigning consecutive integers to the levels of the independent variable or treating the independent variable as a continuous variable as described in the foot notes of each table.

## 4. Results

The subjects examined in the present study are 98 men and 171 women. Their age distributions are shown in Table 1. Sex and age-specific levels of RBC Hg and serum EPA are summarized in Table 1 as well. The RBC-Hg level decreased with age among men (*P* < 0.001) but not among women (*P* = 0.114). Serum concentrations of EPA also decreased with age among men (*P* = 0.001) but less evidently among women (*P* = 0.070). The EPA levels in the HDL fraction of sera were also examined. Age dependence was much less evident and not statistically significant among men or women. 

RBC Hg levels were related to the frequencies of fish consumption in a week (*P* = 0.035) obtained from the interview among women but not among men (*P* = 0.643) as shown in Table 2. However, such a relationship was not observed among men. On the other hand, the serum level of EPA, which is considered to be almost exclusively derived from fish consumption, was not related to fish consumption among men (*P* = 0.207) or women (*P* = 0.582). However, the EPA level in the HDL fraction of the serum increased with fish consumption among both men (*P* = 0.014) and women (*P* = 0.073). 

 The association between the levels of serum EPA and RBC Hg was examined (Table 3). Serum EPA levels were related to RBC mercury levels among women (*P* = 0.005) but not among men (*P* = 0.149). In contrast, serum EPA levels in the HDL fraction of the serum were also significantly related to RBC mercury levels among women (*P* = 0.001) but not among men (*P* = 0.568).

Although the questionnaire used in our interview survey did not include questions of fish species, processing methods (raw fish, fish paste products, or dry fish) were asked. Men tended to consume more dry fish than women did although the difference was not statistically significant (Table 4). 

## 5. Discussions

The total daily amount of mercury intake is known to be strongly related to the amount of fish consumption [12, 13]. The fish consumption, which was measured directly or by using food frequency questionnaires, was related to EPA concentrations in RBC [14–16]. In the present study, RBC Hg concentrations were related to fish consumption among women but not among men. 

Only a limited number of studies examined the association of serum EPA and mercury levels with fish consumption. Innis et al. reported that RBC EPA levels were related to blood mercury levels among children with relatively low mercury levels [17], but this association became unclear when blood mercury levels were 28.9 nmol/L or higher. In the present study, neither among men nor among women, serum EPA level was related to weekly fish consumption even though fish consumption is the only source of serum EPA. However, the EPA level in the HDL fraction of the serum increased with fish consumption in both men and women. The concentrations of triglycerides in plasma increased significantly after the meal and fell below fasting levels by 9 and 12 hours [18]. In the contrast, the major HDL apolipoproteins are apo A-I and apo A-II, and both are required for normal HDL biosynthesis [19]. The turnover of apo A is considered to have a turnover rate of about 4 days [20]. On the other hand, the major protein component of low-density lipoprotein and very-low-density lipoprotein is apolipoprotein B, whose turnover is much shorter than 4 days [21, 22]. Therefore, EPA level in HDL fraction was related more strongly to weekly fish consumption while total serum EPA reflects much more recent dietary intake. Fish consumption was related to only HDL-EPA levels in our study. So we suspected that our questionnaire reflected long-term fish consumption. In addition, the relationship between Hg levels in RBC and HDL EPA levels was stronger than the association of RBC Hg levels with serum EPA levels. After entry, mercury stays on in RBCs during their life span, which is 120 days on average [23–25], and, therefore, Hg levels in RBC may be more strongly related to HDL EPA levels than serum EPA levels. 

Even if the notions described above are correct, it is difficult to explain the sex difference of the association between serum EPA and RBC-Hg levels. Note that, while EPA levels both in the serum and the HDL fraction of the serum were highly significantly related to RBC-Hg levels among women, such an association was not observed among men. According to the survey of household spending, conducted by Japanese Ministry of Agriculture (Forestry and Fisheries white paper 2003), men aged 60 years or older tend to spend more money for eating out than for buying foods and groceries while women spend less for eating out. The types of fish which are consumed in home are saury, yellowtail, and horse mackerels. On the other hand, the types of fish which are consumed in food service industries are more heterogeneous, and they are tuna, shrimp, salmon, and trout, and so on. As shown in Table 4, men tended to eat dry fish more frequently than women. Most typical dry fish foods consumed by Japanese population uses horse mackerel and sardine, which belong to the bottom of the food chain and relatively high in EPA concentration. These observations suggest a possibility that men eat a variety of fish species that may have weak correlations between Hg and EPA contents in fish meats.

The present study was conducted in a southern island in Japan, where fish consumption was considered to be relatively higher than the other areas in Japan. In the present study, geometric means of RBC-Hg level among men and women were 38.6 ng/g and 28.2 ng/g, respectively. They correspond to scalp hair mercury concentrations of 7.0 and 5.1 ppm, which were assuming that (i) the ratio of total mercury concentrations in scalp hair (*μ*g/g) to blood (*μ*g/mL) is 250 [26]; (ii) the ratio between serum and RBC levels of total mercury is 0.264 [27]; (iii) hematocrit is about 40%. Those estimated mercury concentrations in scalp hair are higher than those in other areas of Japan [28], but similar to Wakisaka et al.'s study. They surveyed at the same area [29]. 

The relatively high mercury concentrations among men than among women, observed in the present study, were also reported from studies in Japan [28], probably reflecting sex difference in fish consumption. 

We used wet digestion method to measure total mercury levels. In human, unless occupational exposure to inorganic mercury is present, the major source of mercury is the consumption of fish or fish products, which contain only a small portion of inorganic mercury. Since inorganic mercury is poorly absorbed through digestive tracts, most of mercury in red blood cells is methyl mercury [5]. Another reason for measuring total mercury is its smaller measurement errors when compared to the measurements of methyl mercury and inorganic mercury.

## 6. Limitation

The mercury levels and n3-PUFA levels are different in different fish species. Our question did not include the details of the fish. Our study area is a small community where fishery is one of the major industries. Inhabitants, particularly, the families of fishermen and those working in fishing industry tend to eat locally caught small-sized fish (and its products), which tend to be placed low in the food chain and therefore has relatively low mercury concentrations [30]. On the other hand, as for the rest of inhabitants, they have more opportunity to take mercury-rich fish, such as tunas, because these fish are supplied anywhere and all the year around (Japanese Ministry of Agriculture, Forestry and Fisheries white paper 2003).

 We asked fish frequency. He et al. [31] reported that the fish intake frequency increased the n3-PUFA intake from fish [31]. In other words, it is expected that the fish frequency reflects the amount of fish. We did not use same questionnaire of He. However, it is possible to expect that our fish frequency reflects fish amount to some extent. 

Both methyl mercury and persistent organic pollutants (POPs) are a group of persistent environmental chemicals. They are found in every level of the food chain. Particularly, these chemicals accumulate in humans mostly through fish and shellfish. From the nutritional viewpoint, fish is recommended because it is rich is n3-PUFA. Therefore, from the perspective of risk assessment, the above health hazard issues are particularly important in fish-eating populations [32, 33]. In the present study, we discussed only methyl mercury. It was desirable to have measured POPs.

## 7. Conclusion

The present study showed that fish consumption is not always related to blood mercury levels and EPA. Those findings suggest that the health benefit of fish consumption can be maximized by the careful selection of fish species consumed.

## Figures and Tables

**Table 1:** Age specific concentrations of total mercury, EPA, and HDL-EPA.

	Age				*P* for trend^∗1^
	<69	70–74	75–79	>80	
Hg in red blood cells					
Men					
*N*	26	25	24	23	
Mean^∗2^ (ng/g)	41.9	33.9	28.2	23.7	<0.001
95% CI	33.3–53.4	28.6–40.5	22.2–36.5	17.8–32.4	
Women					
*N*	70	29	30	42	
Mean^∗2^ (ng/g)	24.3	21.7	17.6	21.7	0.114
95% CI	20.6 –29.0	16.4–29.5	13.7–23.0	18.0–26.4	

EPA in sera					
Men					
*N*	26	25	24	23	
Mean^∗2^ (mg/L)	68.3	60.2	56.3	34.4	0.001
95% CI	54.0–87.6	45.3–81.7	42.8–75.5	25.4–48.0	
Women					
*N*	70	29	30	42	
Mean^∗2^ (mg/L)	57.3	57.2	54.5	48.2	0.070
95% CI	51.4–64.1	47.6–69.3	46.1–64.8	41.3–56.6	

EPA in the HDL fraction of sera					
Men					
*N*	26	24	24	21	
Mean^∗2^ (mg/L)	25.0	22.6	26.1	15.9	0.076
95% CI	19.9–32.0	16.8–32.0	20.0–35.6	12.0–21.7	
Women					
*N*	71	30	29	40	
Mean^∗2^ (mg/L)	21.1	23.8	20.8	19.1	0.350
95% CI	18.8–23.7	20.2–28.2	17.1–25.6	16.1–22.9	

^∗1^: The continuous *P* for trend was calculated by treating age as a continuous variable.

^∗2^: Geometric mean.

**Table 2:** RBC mercury concentration and serum EPA and HDL-EPA levels in association with weekly fish consumption.

	Weekly fish consumption^∗1^				
	<2	3-4	5-6	>7	*P* for trend^∗2^
Hg levels in RBC					
Men					
*N*	14	34	23	21	
Mean Hg^∗3^ (ng/g)	50.1	41.7	35.7	51.9	0.643
95% CI	35.0–71.7	32.2–53.8	26.5– 48.3	38.1–70.8	
Women					
*N*	21	79	45	20	
Mean Hg^∗3^ (ng/g)	22.1	25.2	24.1	35.1	0.035
95% CI	15.9–30.7	21.2–30.1	19.5– 29.7	26.1–47.2	

Serum EPA levels					
Men					
*N*	14	34	23	21	
Mean EPA^∗3^ (mg/L)	55.9	74.5	68.3	78.2	0.207
95% CI	36.5–85.8	54.9–101.2	47.8–97.7	54.0– 113.2	
Women					
*N*	21	79	45	20	
Mean EPA^∗3^ (mg/L)	59.0	57.5	60.6	63.2	0.582
95% CI	45.8–75.8	50.7–65.3	52.1–70.4	51.2–78.0	

EPA levels in serum HDL fraction					
Men					
*N*	14	34	23	21	
Mean^∗3^ (mg/L)	21.2	23.5	29.6	33.2	0.014
95% CI	14.1–31.8	17.6–31.5	21.0–41.8	23.3–47.3	
Women					
*N*	21	79	45	20	
Mean^∗3^ (mg/L)	17.9	21.4	22.8	25.0	0.073
95% CI	13.5–23.7	18.6–24.7	19.3–27.0	19.8–31.5	

^∗1^: Raw fish (times/week) + fish-paste products (times/week) + dry-fish (times/week).

^∗2^: When calculating *P* values, age was always included in the models as a covariate. Trend of association was assessed by a regression model assigning consecutive integers to the levels of the weekly fish consumption.

^∗3^: Geometric mean.

**Table 3:** Total mercury concentration (ng/g) in red blood cells by EPA and HDL-EPA levels.

	Men			Women		
	RBC Hg levels			RBC Hg levels		
	*N*	Mean^∗1^ (ng/g)	95% CI	*N*	Mean^∗1^ (ng/g)	95% CI
EPA levels in sera (mg/L)						
<40	26	39.0	28.3–53.8	36	21.2	16.6–27.0
40–	27	33.1	24.6–44.6	55	24.7	20.2–30.4
60–	11	54.7	38.9–76.9	46	27.2	22.1–33.4
80+	33	44.9	34.5–58.4	32	33.1	25.7–42.5
		*P* for trend^∗2^ = 0.149			*P* for trend^∗2^ = 0.005	

EPA levels in HDL fraction of sera (mg/L)						
<15	23	43.8	31.6–60.7	32	21.6	17.0–27.4
15–	26	39.8	29.8–53.2	78	24.2	20.3–28.8
25–	12	45.7	31.1–67.2	30	31.8	24.6–41.0
35+	34	46.1	35.4–60.2	30	35.1	27.2–45.4
		*P* for trend^∗2^ = 0.568			*P* for trend^∗2^ = 0.001	

^∗1^: Geometric mean.

^∗2^: When calculating *P* values, age was always included in the models as a covariate. The continuous *P* for trend was calculated by treating the EPA level as a continuous variable.

**Table 4:** The results of logistic regression analysis regarding the sex difference of the forms of fish consumed.

		Men	Women	OR (95% CI)^∗1^	
	Weekly fish consumption	*N*	*N*		
Raw fish					
	−1	4	14	1 (reference)	
	2+	96	169	2.0 (0.6–6.0)	*P* = 0.230
Fish-paste products					
	−1	76	144	1 (reference)	
	2+	24	39	1.1 (0.6–2.0)	*P* = 0.756
Dry fish					
	−1	54	46	1 (reference)	
	2+	46	65	1.5 (0.9–2.5)	*P* = 0.092

^∗1^: When calculating *P* values, age was always included in the models as a covariate.
